# Fungal signatures in a cadaver and a separated upper limb: insights from a forensic case study

**DOI:** 10.1007/s00253-026-13895-x

**Published:** 2026-06-04

**Authors:** Klaudyna Spychała, Łukasz Szleszkowski, Agata Thannhäuser, Agata Piecuch, Jakub Suchodolski, Rafał Ogórek

**Affiliations:** 1 Department of Mycology and Genetics, Faculty of Biological Sciences, University of Wrocław, Przybyszewskiego 63, Wroclaw, 51148 Poland; 2 Department of Forensic Medicine, Faculty of Medicine, Wroclaw Medical University, Mikulicza-Radeckiego 4, Wrocław, 50345 Poland

**Keywords:** Forensic mycology, Identification, Separated body parts, Fungi, Poland

## Abstract

**Abstract:**

The application of fungi in forensic investigations remains limited, particularly in cases involving dismembered human remains. In this study, we characterized the mycobiome of a decomposing human body and a detached upper limb recovered from different locations using culture-dependent methods. A total of 23 fungal species were identified across four culture media and three incubation temperatures, including 20 species from the body and ten from the upper limb. The dominant taxa included *Yarrowia lipolytica*, *Yarrowia deformans*, *Mucor hiemalis*, and *Geotrichum candidum*. Notably, seven species were shared between the body and the detached limb, indicating partially preserved fungal signatures despite environmental differences. These findings suggest that fungal community profiles may serve as a complementary tool for associating separated human body parts and for assessing postmortem relocation. Additionally, 11 fungal species were identified on human remains for the first time (*Acaulium album*, *Acaulium caviariforme*, *Akanthomyces lecanii*, *Dialonectria ullevolea*, *Fusarium acuminatum*, *Fusarium falsibabinda*, *Fusarium oxysporum*, *Fusarium redolens*, *Fusarium sacchari*, *Linnemannia hyalina*, and *Penicillium clavigerum*), expanding current knowledge of postmortem fungal colonization in temperate climates.

**Key points:**

• *The regional postmortem mycobiome of a corpse and a severed upper limb*

• *Shared fungal species suggest partial preservation of mycobiome signatures*

• *Fungal profiles as potential indicators for the identification of dismembered body parts*

**Graphical abstract:**

**Figure:**
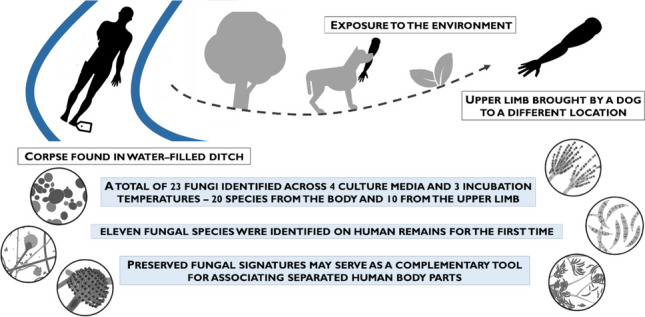

**Supplementary Information:**

The online version contains supplementary material available at 10.1007/s00253-026-13895-x.

## Introduction

Forensic mycology involves the use of fungi or their structures for forensic applications (Hawksworth and Wiltshire 2011). Currently, research on fungi associated with corpses is primarily focused on their potential to assist in postmortem interval (PMI) estimation (Fu et al. 2019; Gemmellaro et al. 2023). The presence of fungi on corpses is a natural phenomenon (Ishii et al. 2006), and their growth can usually be observed after a few days (Spychała et al. 2024). Although fungi are ubiquitous in natural and postmortem environments, their use in forensic investigations has not yet become standard practice (Tranchida et al. 2021). Importantly, there are currently no standardized protocols in forensic mycology, and each research group relies on its own approaches, as already noted by Di Piazza et al. (2021). For this reason, our recent review (Spychała et al. 2024) outlines the fundamental procedures—primarily culture-dependent techniques, including material collection, culture media, and incubation temperature—recommended for use in forensic mycology.

It has already been demonstrated that fungal communities on corpses can vary significantly depending on the stage of decomposition, and that patterns of fungal succession can be observed throughout the decomposition process (Fu et al. 2019; Gemmellaro et al. 2023). The composition and activity of microorganisms on a human corpse are shaped by environmental conditions and fungal adaptations that support their survival and growth. Soil may represent the primary source of microbial communities that drive the decomposition of human remains (Metcalf et al. 2016). Moreover, previous studies have demonstrated that decomposing remains can significantly alter soil microbial communities, including both bacterial and fungal components (Fu et al. 2019; Metcalf et al. 2016; Procopio et al. 2019). Additionally, soil microbial communities associated with decomposing remains represent promising candidates for PMI estimation in cases where bodies are in contact with soil (Procopio et al. 2019). Despite growing interest in forensic mycology, there is still a lack of studies describing the occurrence of fungi on corpses (Tranchida et al. 2018) and studies that would standardize the conclusions reached so far. For this reason, reports on various cases that can contribute to increasing the information available are important.

In forensic practice, cases involving dismemberment and the recovery of separated body parts present a particular challenge (Bilge et al. 2003; Duhing and Martinsen 2007; Maiese et al. 2020). Detached remains may be exposed to different environmental conditions than the main body, including variations in temperature, humidity, substrate, and biotic factors such as animal activity (Franjcevic 2018; Matzen et al. 2022). As a result, post-separation processes may lead to different decomposition pathways and distinct microbial succession patterns on individual body parts. What is more, these environmentally driven differences may complicate the interpretation of biological evidence. At the same time, partially preserved microbial signatures shared between body parts may provide an additional line of evidence for associating separated remains.

Additionally, some species of fungi are characteristic of specific geographical areas. Importantly, environmental conditions or individual characteristics of a given case may influence the mycobiome of a corpse (Di Piazza et al. 2018; Sidrim et al. 2010). It is important to characterize the typical regional postmortem mycobiota, which may contribute to the development of the use of fungi as a forensic tool (Sidrim et al. 2010; Tranchida et al. 2018).

Therefore, the aim of the present study was to characterize the fungal communities colonizing a decomposing human body and a separated upper limb recovered from different locations, and to evaluate the potential of fungal profiles as a tool for linking dismembered body parts and assessing postmortem relocation trajectory.

## Materials and methods

### Case report

The body of an unidentified male in an advanced state of postmortem decomposition was transported to the forensic medicine department in two parts on two separate times in December 2022. First, the right upper limb was delivered. It was discovered on December 8, 2022, on private property after being brought there by the owner’s dog from an unknown location. Following the discovery of these human remains, the next day, as a result of police search operations, a corpse missing its right upper limb from the shoulder joint was discovered in a roadside ditch filled with water. The morphological characteristics of the corpse and remains indicated that they might have come from a single individual, a finding that was unequivocally confirmed by DNA polymorphism testing conducted at the Department of Forensic Medicine (Wrocław Medical University). The identification of DNA fragment polymorphisms was performed using a multiplex test with the PowerPleX Fusion 6C system (Promega, Madison, WI, USA). The examination and autopsy of the body and remains were carried out on December 14, 2022. A hospital identification band was found on the body, and genetic testing confirmed the identity. According to the case file, the man left the hospital on August 11, 2022. Therefore, his body was most likely in the place where it was found for a period of approximately 4 months.

### Sample collection

Samples were collected from the surfaces of the body on December 14, 2022, during the autopsy. Standard microbiological procedures were used to collect the material, i.e., a viscose swab moistened with saline solution (0.85% NaCl, Pol-Aura, Morąg, Poland) was used (Spychała et al. 2024). Eleven swabs were taken from the main parts of the body where mycelium was visible (two swabs per skull and neck; single for torso, side of the abdomen, left arm, back, thighs, calves, feet), as well as two swabs were taken from the separated right upper limb. The samples were stored at 5 ± 0.5 °C (for approximately 3 days) in transport tubes (Spychała et al. 2024) until the fungi were isolated from the material.

### Chemicals

The chemicals used in this study were purchased from the following sources: agar, chloramphenicol, yeast extract (YE), Potato Dextrose Agar (PDA), peptone (Biomaxima, Lublin, Poland), glycerol and glucose (Chempur, Piekary Śląskie, Poland), ox bile (Merck, Darmstadt, Germany), glycerol monostearate (Thermo Fisher Scientific, Waltham, MA, USA), and Tween 60 (Pol-Aura, Morąg, Poland). Commercial olive oil was used as a lipid substrate. DNA was isolated using the Bead-Bead Micro AX Gravity Kit (A&A Biotechnology, Gdańsk, Poland), and PCR products were purified using the Clean-Up Kit (A&A Biotechnology, Gdańsk, Poland). Primers were supplied by Genomed (Warszawa, Poland).

### Isolation of fungi from samples

In order to isolate fungi from the collected material, the swab tips were aseptically cut off and placed in sterile conical polypropylene tubes (25 mL) with caps (FL Medical, Torreglia, Italy) containing 10 mL of saline solution. The tubes were shaken at room temperature for about 5 min to facilitate the release of fungal cells into the solution, allowing for consistent distribution of the test material. Next, 100 µL of the suspension was spread on plates with media in three replicates.

To ensure the recovery of a broad spectrum of fungal species, four different culture media were utilized:

- Modified Leeming–Notman agar (MLNA) (consisting of 2% olive oil, 1% peptone, 1% glycerol, 1% glucose, 0.8% ox bile, 0.5% Tween 60, 0.2% YE, 0.05% glycerol monostearate, and 1.5% agar) for the isolation of *Malassezia* spp.
- Potato Dextrose Agar (PDA)
- Sabouraud’s Glucose Agar (SGA) (consisting of 4% glucose, 1% peptone, and 1.5% agar)
- Yeast Peptone Glucose (YPG) medium (consisting of 2% glucose, 2% peptone, 1% YE, and 2% agar) reduced to 4.5 pH and containing chloramphenicol (50 mg·L^−1^) to inhibit the growth of bacteria and some filamentous fungi (YPG pH 4.5) according to Spychała et al. (2024)

The samples were incubated for 7 to 30 days at temperatures of 5 ± 0.5 °C, 24 ± 0.5 °C, and 37 ± 0.5 °C. Incubation at three different temperatures using four different culture media was intended to isolate the most possible wide range of microscopic culture fungi from the corpses.

Pure cultures were then obtained on PDA medium used for filamentous fungi and YPG medium for yeast-like fungi. In addition, the isolates were preserved on PDA medium (filamentous fungi) and in glycerol (Chempur, Piekary Śląskie, Poland) at − 70 ± 0.1 °C (yeast-like fungi).

### Fungal identification

Fungal identification was performed using a combination of conventional and molecular approaches, as described by Spychała et al. (2025). Phenotypic assessment was performed on PDA medium (for all species). In addition, isolates from the genera *Penicillium* spp. and *Aspergillus* spp. were also assessed on Czapek yeast autolysate agar (CYA; 30.0 g·L^−1^ sucrose, 15 g·L^−1^ agar, 5.0 g·L^−1^ yeast extract, 3.0 g·L^−1^ NaNO_3_, 1.0 g·L^−1^ K_2_HPO_4_, 0.5 g·L^−1^ KCl, 0.5 g·L^−1^ MgSO_4_·7H_2_O, and 0.01 g·L^−1^ FeSO_4_·7H_2_O) and malt extract agar (MEA; BioMaxima, Lublin, Poland). In filamentous fungi, macromorphological features such as colony texture (e.g., velvety, floccose, powdery), pigmentation (surface and reverse), colony diameter, and growth rate were evaluated. Micromorphological characteristics, including conidiophore structure and branching patterns, as well as the shape and size of conidia or sporangia, were also examined. For yeast isolates, phenotypic characterization included colony morphology, cell shape and size, budding pattern, and the formation of pseudohyphae or true hyphae. Additional traits such as growth at different temperatures were considered when applicable. Identification was supported by diagnostic keys and standard mycological monographs (Crous et al. 2021; de Hoog and Smith 2004; Gräfenhan et al. 2011; Kepler et al. 2017; Leslie and Summerell 2006; Samson et al. 2010; Samuels et al. 2002; Sandoval-Denis et al. 2016; Schipper 1978; van der Walt and von Arx 1980; Vandepol et al. 2020; Varga et al. 2011). Genomic DNA from freshly grown fungal cultures was extracted using the Bead-Beat Micro AX Gravity kit (A&A Biotechnology, Gdańsk, Poland) in accordance with the manufacturer’s protocol. Polymerase chain reaction (PCR) was carried out with two pairs of fungal-specific primers: (1) ITS1 (5′-TCCGTAGGTGAACCTGCGG-3′) and ITS4 (5′-TCCTCCGCTTATTGATGC-3′) targeting the internal transcribed spacer (ITS) region of filamentous fungi (White et al. 1990) and (2) NL-1 (5′-GCATATCAATAAGCGGAGGAAAAG-3′) and NL-4 (5′-GGTCCGTGTTTCAAGACGG-3′) amplifying the D1/D2 domain of the LSU rDNA gene in yeast-like fungi (Kurtzman and Robnett 1997). PCR amplification was performed using a T100 thermocycler (Bio-Rad, Berkeley, CA, USA) following the protocols described by White et al. (1990) and Kurtzman and Robnett (1997), respectively. Amplicons were purified with the Clean-Up Kit (A&A Biotechnology, Gdańsk, Poland) and sequenced by Macrogen Europe (Amsterdam, Netherlands; http://dna.macrogen.com/eng/).

### Data analysis

Raw sequences were processed and analyzed using the BioEdit Sequence Alignment Editor (Hall 1999). The resulting PCR amplicons were compared against sequences deposited in the GenBank database of the National Center for Biotechnology Information (NCBI, Bethesda, Rockville, MD, USA) using the BLAST algorithm for taxonomic identification. Verified sequences were subsequently submitted to GenBank. All fungal isolates were deposited in the culture collection of the Department of Mycology and Genetics, University of Wrocław.

To assess the distribution and recovery of fungal species associated with the main body and the detached upper limb at each sampling site, fungal growth was evaluated on four different culture media. Species were recorded as present or absent on each medium, and the total number of positive cultures was used as an indicator of their relative occurrence.

## Results

### Mycological analyses

The human body recovered from a partially flooded ravine approximately 4 months postmortem, along with a detached upper limb displaced from the original deposition site by animal activity, was in an advanced stage of decomposition, with extensive soft tissue loss and partial skeletonization, and showed features consistent with prolonged environmental exposure. A detailed description of the autopsy findings is provided in Supplementary File S1.

Mycological analyses were performed separately for the main part of the body and the separated right upper limb in order to compare the fungi colonizing them. The fungal isolates were divided into groups based on differences in macro- and micromorphology, and one representative of each group was selected for further genetic identification. This yielded 51 isolates from the main part of the body and 16 isolates from the upper limb. Genetic analyses identified 20 species from the body (isolates from UWR_795 to UWR_845) and ten species from the right upper limb (isolates from UWR_846 to UWR_861). Most of the species isolated in the study belonged to the *Ascomycota* phylum, with the exception of isolate UWR_817 belonging to *Mortierellomycota* and isolates UWR_818-UWR_820, UWR_852-UWR_855 which belong to *Mucoromycota* phylum (Tables 1 and 2). Isolates from the body belonged to eight families, while isolates from the upper limb belonged to five families.

**Table 1:** Fungi isolated from cadaver and results of BLAST (all *E* values were zero)

Fungi isolated from cadaver						Identity with sequence from GenBank^1^		
Isolate number	Identified species	Phylum	Family	GenBank accession no	The sequence length (bp)	Query cover, %	Identity, %	Accession
UWR_795	*Acaulium album*	*Ascomycota*	*Microascaceae*	PX651155	404	100	99.51	KY852471.1
UWR_796	*Acaulium caviariforme*	*Ascomycota*	*Microascaceae*	PX651156	392	100	98.98	MW031220.1
UWR_797				PX651157	452	100	98.46	MW031220.1
UWR_798	*Akanthomyces lecanii*	*Ascomycota*	*Cordycipitaceae*	PX651158	521	100	100	MZ544564.1
UWR_799	*Aspergillus niger*	*Ascomycota*	*Aspergillaceae*	PX651159	510	100	100	MN195121.1
UWR_800	*Dialonectria ullevolea*	*Ascomycota*	*Nectriaceae*	PX651187	522	100	100	MH866681.1
UWR_801				PX651188	479	100	100	MH866681.1
UWR_802				PX651189	373	100	100	MH866681.1
UWR_803				PX651190	367	100	99.73	MH866681.1
UWR_804	*Fusarium acuminatum*	*Ascomycota*	*Nectriaceae*	PX651160	352	100	100	OP897712.1
UWR_805	*Fusarium falsibabinda*	*Ascomycota*	*Nectriaceae*	PX651161	370	100	99.73	OR583001.1
UWR_806				PX651162	370	100	99.73	OR583001.1
UWR_807	*Fusarium oxysporum*	*Ascomycota*	*Nectriaceae*	PX651163	398	100	100	PV477281.1
UWR_808	*Fusarium redolens*	*Ascomycota*	*Nectriaceae*	PX651164	450	100	99.78	EF495234.1
UWR_809	*Fusarium sacchari*	*Ascomycota*	*Nectriaceae*	PX651165	464	100	100	PX246651.1
UWR_810	*Fusarium solani*	*Ascomycota*	*Nectriaceae*	PX651166	408	100	100	OR478207.1
UWR_811				PX651167	381	100	100	PP421964.1
UWR_812				PX651168	422	100	100	MT032593.1
UWR_813	*Fusicolla aquaeductuum*	*Ascomycota*	*Nectriaceae*	PX651191	502	100	100	MH087221.1
UWR_814				PX651192	482	100	100	MH087221.1
UWR_815				PX651193	440	100	100	MH087221.1
UWR_816	*Geotrichum candidum*	*Ascomycota*	*Dipodascaceae*	PX651194	506	100	100	KX364934.1
UWR_817	*Linnemannia hyalina*	*Mortierellomycota*	*Mortierellaceae*	PX651169	520	100	100	MH855527.1
UWR_818	*Mucor circinelloides*	*Mucoromycota*	*Mucoraceae*	PX651170	431	100	100	LC413614.1
UWR_819	*Mucor hiemalis*	*Mucoromycota*	*Mucoraceae*	PX651171	558	100	100	MT626048.1
UWR_820				PX651172	572	100	100	MT626048.1
UWR_821	*Penicillium clavigerum*	*Ascomycota*	*Aspergillaceae*	PX651173	493	100	100	MK450681.1
UWR_822	*Trichoderma atroviride*	*Ascomycota*	*Hypocreaceae*	PX651174	490	100	100	KY073424.1
UWR_823	*Yarrowia deformans*	*Ascomycota*	*Dipodascaceae*	PX651195	365	100	99.45	MK394171.1
UWR_824				PX651196	419	100	99.76	MK394171.1
UWR_825	*Yarrowia lipolytica*	*Ascomycota*	*Dipodascaceae*	PX651197	358	100	99.72	LC496593.1
UWR_826				PX651198	364	100	99.73	KY110196.1
UWR_827				PX651199	364	100	99.45	ON202634.1
UWR_828				PX651200	351	100	100	ON606260.1
UWR_829				PX651201	358	100	99.72	MZ391070.1
UWR_830				PX651202	360	100	99.72	LC496593.1
UWR_831				PX651203	364	100	100	MT151658.1
UWR_832				PX651204	389	100	100	LC496593.1
UWR_833				PX651205	425	100	100	LC496593.1
UWR_834				PX651206	359	100	99.72	MZ391070.1
UWR_835				PX651207	362	100	99.72	LC496593.1
UWR_836				PX651208	360	100	100	ON202632.1
UWR_837				PX651209	436	100	100	LC496593.1
UWR_838				PX651210	374	100	100	MT151658.1
UWR_839				PX651211	350	100	100	ON202634.1
UWR_840				PX651212	404	100	99.75	MT151658.1
UWR_841				PX651213	359	100	99.72	MK358177.1
UWR_842				PX651214	383	100	100	OR084802.1
UWR_843				PX651215	418	100	99.76	ON838587.1
UWR_844				PX651216	363	100	99.72	MT151658.1
UWR_845				PX651217	485	100	99.79	LC496593.1

^1^Sequences from UWR_795 to UWR_799, from UWR_804 to UWR_812, and from UWR_817 to UWR_822 were obtained using the ITS1 and ITS4 primer pair, while sequences from UWR_800 to UWR_803, from UWR_813 to UWR_816, and from UWR_823 to UWR_845 were obtained using a pair of primers NL-1 and NL-4

**Table 2:** Fungi isolated from separated right upper limb and results of BLAST (all *E* values were zero)

Fungi isolated from right upper limb						Identity with sequence from GenBank^1^		
Isolate number	Identified species	Phylum	Family	GenBank accession no	The sequence length (bp)	Query cover, %	Identity, %	Accession
UWR_846	*Acaulium album*	*Ascomycota*	*Microascaceae*	PX651175	457	100	99.56	OP597822.1
UWR_847	*Dialonectria ullevolea*	*Ascomycota*	*Nectriaceae*	PX651176	373	100	100	MH855229.1
UWR_848	*Fusarium solani*	*Ascomycota*	*Nectriaceae*	PX651177	469	100	99.36	MW165528.1
UWR_849				PX651178	385	100	100	MN535822.1
UWR_850	*Fusicolla aquaeductuum*	*Ascomycota*	*Nectriaceae*	PX651218	465	100	100	MH087221.1
UWR_851	*Geotrichum candidum*	*Ascomycota*	*Dipodascaceae*	PX651219	486	100	100	MN197675.1
UWR_852	*Mucor flavus*	*Mucoromycota*	*Mucoraceae*	PX651179	582	100	99.83	JN206061.1
UWR_853	*Mucor hiemalis*	*Mucoromycota*	*Mucoraceae*	PX651180	557	100	100	MT366055.1
UWR_854				PX651181	517	100	100	MT626048.1
UWR_855	*Mucor mucedo*	*Mucoromycota*	*Mucoraceae*	PX651182	356	100	99.72	MH854783.1
UWR_856	*Penicillium griseofulvum*	*Ascomycota*	*Aspergillaceae*	PX651183	354	100	100	MN545450.1
UWR_857	*Yarrowia lipolytica*	*Ascomycota*	*Dipodascaceae*	PX651220	353	100	100	MT151658.1
UWR_858				PX651221	381	100	100	LC496593.1
UWR_859				PX651222	355	100	99.72	LC496593.1
UWR_860				PX651223	362	100	99.72	MT151658.1
UWR_861				PX651224	425	100	100	OR084802.1

^1^Sequences from UWR_846 to UWR_849 and from UWR_852 to UWR_856 were obtained using the ITS1 and ITS4 primer pair, while sequences UWR_850, UWR_851, and from UWR_857 to UWR_861 were obtained using the NL-1 and NL-4 primer pair

All fungal rDNA nucleotide sequences obtained in this study are publicly available in the GenBank database under accession numbers PX651155 through PX651174 (sequences amplified using the ITS1/ITS4 primer pair) and sequences with accession numbers PX651187-PX651217 (obtained using the NL-1/NL-4 primer pair) for isolates found in cadavers (Table 1). In contrast, isolates obtained from the right upper limb were deposited under numbers PX651175-PX651183 (using the ITS1/ITS4 primer pair) and PX651218-PX651224 (amplified using NL-1/NL-4) (Table 2).

Based on BLAST analysis, all sequences had an *E* value of zero, and 100% query cover. The sequence lengths ranged from 350 to 572 bp and identity range of 98.46–100% for isolates from the cadaver. The sequence lengths obtained from isolates inhabiting right upper limb ranged from 353 to 582 bp, and their identity values ranged from 99.36 to 100% (Tables 1 and 2).

A total of 23 fungal species were identified throughout the study—20 species on the body and ten on the right upper limb. Importantly, seven species colonized both the body and the separated right upper limb. Thirteen species were found exclusively on the body, while three species were found on the upper limb but not on the body.

**Fig. 1:**
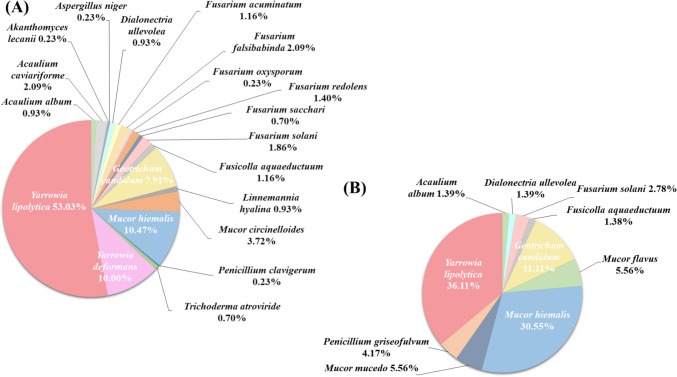
Percentage distribution of all fungal species isolated from the body (**A**) and from the separated right upper limb (**B**)

The dominant species found on the body was *Yarrowia lipolytica* (53.03%), followed by *Mucor hiemalis* (10.47%), *Yarrowia deformans* (10%), and *Geotrichum candidum* (7.91%) (Fig. 1A). In the case of the right upper limb, corresponding species were also the most frequent, namely *Y. lipolytica* (36.11%), *M. hiemalis* (30.55%), and *G. candidum* (11.11%) (Fig. 1B).

A comprehensive list of fungal isolates, organized by sampling location and culture conditions, is presented in Tables 3 and 4. In the case of the body, the largest number of species was recorded on the left hand (11 species) and on the torso (nine species), while only four species of fungi were isolated from the side of the abdomen and calves. In the case of the separated right upper limb, a similar number of species was isolated from both samples (six species from the arm and forearms and eight species from the right hand). Three species were isolated from the body only once (i.e., *Akanthomyces lecanii*, *Aspergillus niger*, and *Penicillium clavigerum*).

**Table 3:** Cultures of fungi isolated from a cadaver with information on material collection site (numbers), temperature during cultivation, and culture medium

Isolate number	Species^1^	5 °C				24 °C				37 °C			
		YPG ^pH 4.5^	PDA	SGA	MLNA	YPG ^pH 4.5^	PDA	SGA	MLNA	YPG ^pH 4.5^	PDA	SGA	MLNA
UWR_795	*Acaulium album*	+ ^2, 6, 9^	+ ^6^										
UWR_796	*Acaulium caviariforme*	+ ^2, 9^											
UWR_797		+ ^4, 5, 7, 8, 9^	+ ^8^		+ ^8^								
UWR_798	*Akanthomyces lecanii*						+ ^1^						
UWR_799	*Aspergillus niger*										+ ^9^		
UWR_800	*Dialonectria ullevolea*	+ ^9^											
UWR_801			+ ^3^										
UWR_802					+ ^5^								
UWR_803					+ ^5^								
UWR_804	*Fusarium acuminatum*		+ ^3^			+ ^1^				+ ^1^	+ ^1^		+ ^1^
UWR_805	*Fusarium falsibabinda*					+ ^3^	+ ^3^	+ ^3^	+ ^3^		+ ^3^	+ ^3^	+ ^3^
UWR_806						+ ^3^			+ ^3^				
UWR_807	*Fusarium oxysporum*						+ ^3^						
UWR_808	*Fusarium redolens*					+ ^1^	+ ^1^			+ ^2^	+ ^1^	+ ^1, 2^	
UWR_809	*Fusarium sacchari*						+ ^3^			+ ^3^		+ ^3^	
UWR_810	*Fusarium solani*							+ ^7^				+ ^7^	
UWR_811								+ ^2^		+ ^2, 5^	+ ^2^	+ ^2^	
UWR_812												+ ^6^	
UWR_813	*Fusicolla aquaeductuum*		+ ^3^										
UWR_814					+ ^5^								
UWR_815		+ ^5^	+ ^5^	+ ^5^									
UWR_816	*Geotrichum candidum*	+ ^9^				+ ^1, 4, 5, 7, 8, 9^	+ ^1, 4, 6, 8^	+ ^6, 9^	+ ^5, 8^	+ ^1, 4, 6, 7, 8, 9^	+ ^4, 5, 6, 8, 9^	+ ^1, 4, 8, 9^	+ ^5, 6, 8, 9^
UWR_817	*Linnemannia hyalina*	+ ^1^	+ ^1^	+ ^1^	+ ^1^								
UWR_818	*Mucor circinelloides*	+ ^2, 5^	+ ^2^	+ ^2, 5^	+ ^2^	+ ^2, 5^	+ ^2^			+ ^2, 5^	+ ^2, 5^	+ ^2^	+ ^2, 5^
UWR_819	*Mucor hiemalis*		+ ^6^										
UWR_820		+ ^1, 2, 6, 7, 9^	+ ^1, 2, 6, 7, 9^	+ ^1, 9^	+ ^1, 2, 7, 9^	+ ^1, 2, 6, 7, 9^	+ ^1, 2, 6, 7, 9^	+ ^6, 9^	+ ^1, 2, 9^	+ ^1, 6^	+ ^1, 2, 6, 7, 9^	+ ^1, 6, 9^	+ ^1, 2, 9^
UWR_821	*Penicillium clavigerum*		+ ^5^										
UWR_822	*Trichoderma atroviride*						+ ^5^	+ ^5^			+ ^5^		
UWR_823	*Yarrowia deformans*	+ ^3, 5, 7, 8^	+ ^3, 5, 7, 8^	+ ^3, 5, 8^	+ ^3, 5, 7, 8^								
UWR_824		+ ^2, 4, 6^	+ ^2, 4, 6^	+ ^2, 4^	+ ^2, 4, 6^	+ ^4, 6^	+ ^4, 6^	+ ^4, 6^	+ ^3, 4, 6^	+ ^4, 6^	+ ^4, 6^	+ ^4, 6^	+ ^2, 4, 6^
UWR_825	*Yarrowia lipolytica*	+ ^1^	+ ^1^	+ ^1^	+ ^1^								
UWR_826					+ ^5^								
UWR_827										+ ^5^			
UWR_828		+ ^3, 4, 8^	+ ^3, 4, 8^	+ ^3, 4, 8^	+ ^3, 4, 8^								
UWR_829						+ ^8^	+ ^8^	+ ^8^	+ ^8^	+ ^8^	+ ^8^	+ ^8^	+ ^8^
UWR_830											+ ^5^		
UWR_831		+ ^9^	+ ^9^		+ ^9^								
UWR_832						+ ^9^							+ ^11^
UWR_833		+ ^2^	+ ^2^		+ ^2^		+ ^2^	+ ^2^		+ ^2^	+ ^2^	+ ^2^	
UWR_834						+ ^9^	+ ^9^	+ ^9^	+ ^9^	+ ^9^	+ ^9^	+ ^9^	+ ^9^
UWR_835									+ ^3^				
UWR_836										+ ^2^			+ ^2^
UWR_837		+ ^3^	+ ^3^	+ ^3^	+ ^3^	+ ^1, 3^	+ ^1, 3^	+ ^3^	+ ^3^	+ ^3^	+ ^3^		+ ^3^
UWR_838		+ ^2^	+ ^2^	+ ^2^	+ ^2, 6^	+ ^2,^	+ ^6^	+ ^6^	+ ^4, 6^	+ ^2, 6^	+ ^6^	+ ^6^	+ ^2, 6^
UWR_839						+ ^3, 8, 9^	+ ^3, 8, 9^	+ ^1, 3, 8, 9^	+ ^3, 8, 9^	+ ^1, 5, 8, 9^	+ ^3, 8, 9^	+ ^1, 3, 8, 9^	+ ^2, 3, 8, 9^
UWR_840						+ ^3^	+ ^3^	+ ^3^	+ ^3^	+ ^3^	+ ^3^	+ ^3^	+ ^3^
UWR_841		+ ^5, 8^	+ ^5, 8^	+ ^5, 8^	+ ^8^	+ ^1, 3, 4^	+ ^1, 3, 4^	+ ^3, 4^	+ ^3, 4^	+ ^3, 4, 6^	+ ^3, 4^	+ ^3, 4^	+ ^3, 4, 6^
UWR_842				+ ^2^						+ ^2, 7^	+ ^7^		+ ^7^
UWR_843		+ ^2, 6^	+ ^2, 6^		+ ^2, 6^	+ ^2, 3, 4, 5, 6, 8, 9^	+ ^2, 4, 5, 6, 8, 9^	+ ^1, 2, 4, 5, 6, 8, 9^	+ ^2, 3, 4, 5, 6, 8, 9^	+ ^1, 2, 4, 6, 8, 9^	+ ^1, 2, 4, 6, 8, 9^	+ ^1, 2, 4, 5, 6, 8, 9^	+ ^1, 2, 4, 6, 8, 9^
UWR_844		+ ^7^	+ ^7^		+ ^7^	+ ^3^	+ ^3, 7^	+ ^3, 7^	+ ^3, 7, 9^	+ ^7^	+ ^3, 7^	+ ^3, 7^	+ ^3, 7, 9^
UWR_845		+ ^7^	+ ^7^		+ ^7^								

^1^A “+” sign indicates that the respective fungal strain has been isolated from a specific site. Labelling of individual material collection sites: 1, skull; 2, neck; 3, torso; 4, side of the abdomen; 5, left hand; 6, back; 7, thighs; 8, calves; 9, feet

*YPG pH 4.5* yeast peptone glucose medium reduced to 4.5 pH and containing chloramphenicol), *PDA* potato dextrose agar, *SGA* sabouraud’s glucose agar, *MLNA* modified leeming–notman agar

**Table 4:** Cultures of fungi isolated from a right upper limb of a corpse with information on material collection site (numbers), temperature during cultivation, and culture medium

Isolate number	Species^1^	5 °C			24 °C				37 °C				
		YPG ^pH 4.5^	PDA	SGA	MLNA	YPG ^pH 4.5^	PDA	SGA	MLNA	YPG ^pH 4.5^	PDA	SGA	MLNA
UWR_846	*Acaulium album*	+ ^1^											
UWR_847	*Dialonectria ullevolea*		+ ^1^										
UWR_848	*Fusarium solani*											+ ^2^	
UWR_849												+ ^2^	
UWR_850	*Fusicolla aquaeductuum*	+ ^2^											
UWR_851	*Geotrichum candidum*					+ ^1^	+ ^1^			+ ^1^	+ ^1, 2^	+ ^1, 2^	+ ^1^
UWR_852	*Mucor flavus*	+ ^1^	+ ^2^	+ ^2^	+ ^2^								
UWR_853	*Mucor hiemalis*	+ ^2^	+ ^2^	+ ^2^	+ ^2^								
UWR_854		+ ^1, 2^	+ ^2^	+ ^2^	+ ^1, 2^	+ ^1, 2^	+ ^1, 2^	+ ^1^	+ ^1^	+ ^1, 2^	+ ^1, 2^	+ ^1^	+ ^1^
UWR_855	*Mucor mucedo*	+ ^2^	+ ^2^	+ ^2^	+ ^2^								
UWR_856	*Penicillium griseofulvum*		+ ^2^	+ ^2^	+ ^2^								
UWR_857	*Yarrowia lipolytica*	+ ^1^	+ ^1^	+ ^1^	+ ^1^	+ ^2^	+ ^2^			+ ^2^		+ ^2^	+ ^2^
UWR_858										+ ^1^	+ ^2^	+ ^1, 2^	
UWR_859			+ ^2^	+ ^2^	+ ^2^								
UWR_860						+ ^1^	+ ^1^	+ ^1^	+ ^1^	+ ^1^	+ ^1^	+ ^1^	+ ^1^
UWR_861										+ ^1^		+ ^1^	

^1^A “ + ” sign indicates that the respective fungal strain has been isolated from a specific site. Labelling of individual material collection sites: 1, upper arm + forearm; 2, right hand

*YPG pH 4.5* yeast peptone glucose medium reduced to 4.5 pH and containing chloramphenicol, *PDA* potato dextrose agar, *SGA* sabouraud’s glucose agar, *MLNA* modified leeming–notman agar

**Fig. 2:**
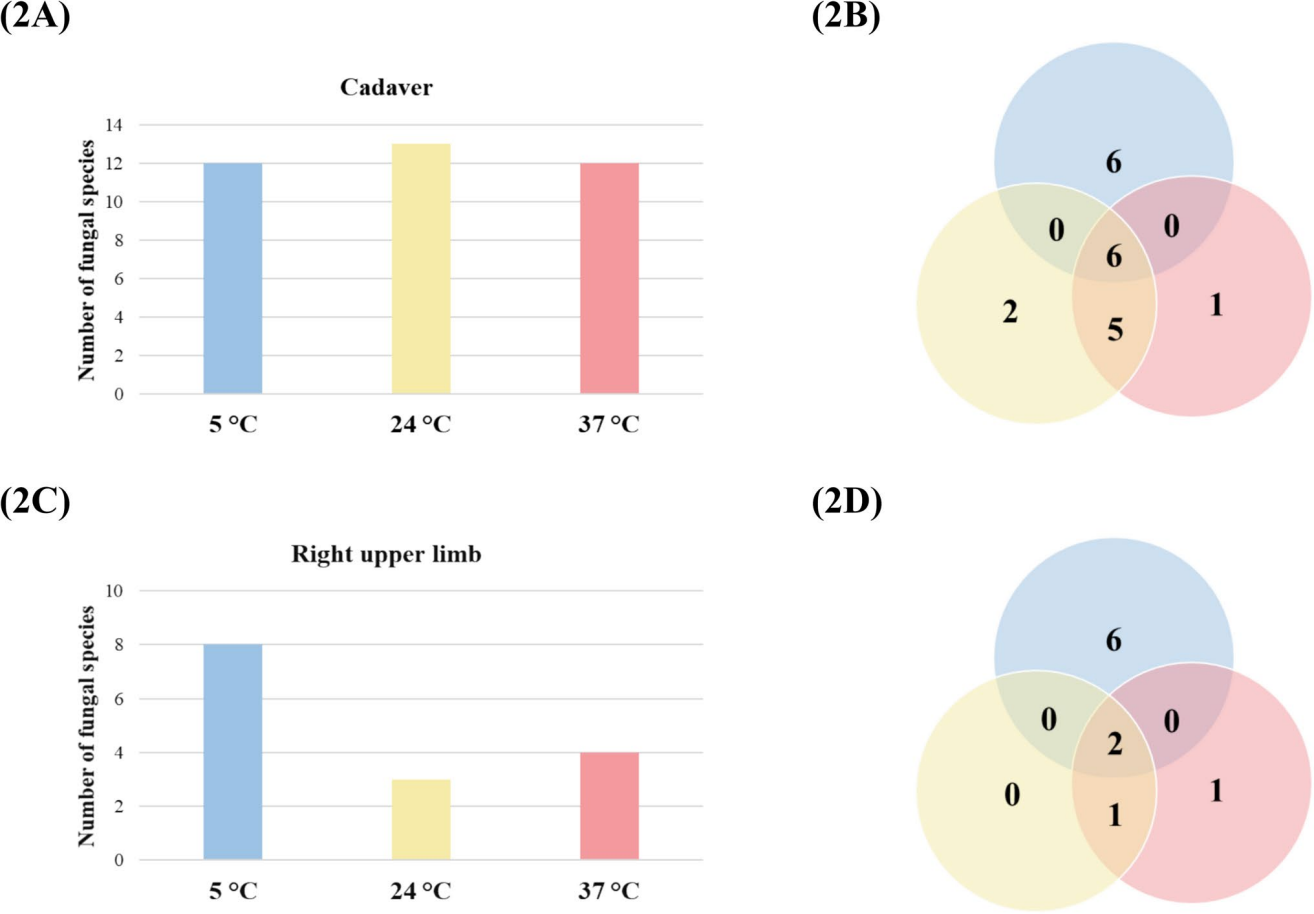
Fungal species identified at 5 °C, 24 °C, and 37 °C in samples from the cadaver (**A**) and the separated right upper limb (**C**). Venn diagrams (**B**, **D**) present the distribution of shared and temperature-specific species within each sample

In the body samples, the highest diversity was observed at 24 °C—although similar numbers of fungal species were isolated at the other temperatures. In contrast, in the samples from the right upper limb, the highest diversity was observed at 5 °C (Fig. 2).

## Discussion

Forensic mycology is a developing field within forensic sciences, and the application of fungi has been proposed in various contexts, including PMI estimation and microtrace evidence analysis (Hawksworth and Wiltshire 2011). However, the limited number of studies of the postmortem mycobiome makes it difficult to draw strong conclusions about its forensic utility. Large-scale mycological analyses of human remains would be valuable for understanding variability, but such studies are often constrained by legal and ethical considerations. Consequently, well-documented case studies remain an important source of data. However, such studies should be interpreted as exploratory and hypothesis-generating rather than definitive evidence of reproducible forensic patterns.

Nevertheless, here, we present the mycological assessment of a decomposed human body found in a partially flooded ravine, approximately 4 months postmortem, together with a detached upper limb that had been displaced to a different location by animal activity. Given the complexity of such postmortem scenarios and the potential variability of fungal communities, a broad methodological approach was required.

### Culture conditions and methodological considerations

In order to capture the widest possible spectrum of fungal species using culture-dependent methods, multiple culture media are necessary to accommodate the diverse growth requirements of different fungal groups (Abdullah and Saadullah 2018; Korkom et al. 2025). Therefore, the present study was conducted in accordance with the protocol proposed by Spychała et al. (2024) by applying four culture media widely used in mycology. Sabouraud agar and PDA support the growth of a wide variety of environmental fungi, and indeed, the highest number of isolates was obtained using these media. YPG medium, supplemented with chloramphenicol and adjusted pH ~ 4.5, allows for recovery of yeast-like fungi by inhibiting the growth of fast-growing filamentous species (Spychała et al. 2024). However, here, several mold species were also successfully cultivated on YPG. During body decomposition, lipid degradation provides substrates for lipophilic fungi such as *Malassezia* spp., which are part of the normal skin mycobiome of living hosts (Juntachai et al. 2009; Caballero-Moreno et al. 2024). Thus, a MLNA medium, designed for isolating *Malassezia* spp., was employed. Nevertheless, since this medium is non-selective and supports the growth of molds and other yeasts, several non-*Malassezia* species were obtained on this medium during this study.

The above findings highlight that, under postmortem conditions, fungal growth patterns do not strictly follow canonical cultivation expectations (Abdullah and Saadullah 2018). Instead, considerable overlap in growth across different media was observed, reflecting the ecological flexibility of postmortem fungal communities. Thus, our results support the need for multi-medium approaches in forensic mycology to avoid underestimation of fungal diversity. However, the use of multiple culture media may have introduced variability in fungal recovery and isolate selection. Microscopic fungi exhibit phenotypic differences depending on the culture medium, and different taxa may display similar colony morphologies (Samson et al. 2010). Consequently, the selection of representative colonies for further analysis may have resulted in the underrepresentation of low-abundance or morphologically similar taxa. In order to minimize this limitation, culture media were standardized for each individual isolate during the morphological analyses.

Although exogenous contamination cannot be entirely excluded, all procedures were performed under conditions aimed at minimizing contamination and preserving the original fungal profile of the cadaver. In addition, the presence of biologically plausible fungal communities suggests that the obtained results reflect the actual mycobiome associated with the cadaver.

### Characteristics of fungal species previously unreported from human remains

While many of the isolated species have previously been reported on decomposing remains, several taxa identified in this study have not, to our knowledge, been described in this context before. These include the following: *Acaulium album*, *Acaulium caviariforme*, *A. lecanii*, *Dialonectria ullevolea*, *Fusarium acuminatum*, *Fusarium falsibabinda*, *Fusarium oxysporum*, *Fusarium redolens*, *Fusarium sacchari*, *L. hyalina*, and *P. clavigerum*.

*A. album* is commonly found in Europe and North America on decomposing organic materials and various types (often carnivore) of feces (Woudenberg et al. 2017), while *A. caviariforme* was isolated from the body of a dead dormouse found in a cave (Nováková et al. 2018). *A. lecanii* is a well-known entomopathogen and hyperparasite of some rusts (Nana et al. 2023). All of the listed species of the genus *Fusarium* are plant pathogens (Olszak-Przybyś et al. 2023; Yu et al. 2024; Wang et al. 2022; Viswanathan et al. 2017), although *F. oxysporum* can also infect humans (Nag et al. 2022). *D. ullevolea* is often isolated from deciduous trees (Gräfenhan et al. 2011). *P. clavigerum* is a saprotroph that produces characteristic secondary metabolites (Frisvad and Samson 2004). Interestingly, the presence of *Fusicolla aquaeductuum*, already reported in forensic contexts and commonly associated with aquatic habitats (Beaulieu et al. 2025; Gräfenhan et al. 2011), may reflect the deposition of the body in a water-filled environment, whereas taxa such as *L. hyalina* or *Trichoderma atroviride* are consistent with soil-derived colonization (Tseng et al. 2022; Traversari et al. 2024).

### Temperature as a factor shaping fungal diversity

This ecological flexibility is further reflected in the wide range of temperature requirements exhibited by fungi colonizing decomposing remains. Fungi associated with corpses may be of external or internal origin and therefore exhibit diverse temperature requirements (Spychała et al. 2024). Therefore, three incubation temperatures were applied to capture a broad spectrum of taxa. Most fungi from both the limb and cadaver samples were isolated across all temperatures. However, several species were recorded exclusively at 5 °C, including psychrotolerant taxa such as *Linnemannia hyalina*, *P. clavigerum*, and *Mucor flavus* (Telagathoti et al. 2021; Frisvad and Samson 2004; Danilova et al. 2024), as well as members of the genus *Acaulium*, which are capable of growth at low temperatures (Sandoval-Denis et al. 2016). In temperate climates during autumn and winter, lower ambient temperatures favor the development of psychrotolerant or psychrotrophic fungi. The average temperature at the recovery site was approximately 5 °C in November and 3 °C in December (https://www.weather-atlas.com/en/poland/wroclaw-climate. Accessed April 13, 2026), which likely favored the presence of such taxa. On the other hand, prolonged exposure of the remains to low temperatures may have also shaped the fungal community by limiting temperature-sensitive species. Notably, no species of *Malassezia* were recovered despite the use of selective media, which may reflect their limited viability under conditions around 4 °C (Crespo et al. 2000).

This highlights that the interpretation of fungal evidence requires consideration of multiple environmental influences beyond temperature alone. In cases involving dismemberment, detached body parts may be exposed to distinct environmental conditions, leading to divergent decomposition pathways and microbial succession patterns (Franjcevic 2018; Matzen et al. 2022). The present findings reflect this complexity, as the majority of the identified taxa are typical environmental fungi, suggesting a substantial contribution of external sources to the observed mycobiome.

### Environmental influence on postmortem fungal communities

The ecological characteristics of the identified taxa strongly support their predominantly external origin and highlight the influence of the surrounding environment on the postmortem mycobiome. This is particularly evident in species associated with specific ecological niches. These patterns become more apparent when comparing the cadaver and the detached limb. Although the higher number of taxa unique to the cadaver may partially reflect the larger number of collected samples, the detected species, including *F. acuminatum*, *F. oxysporum*, *F. redolens*, and *L. hyalina*, nevertheless suggest more extensive and sustained interaction with the surrounding environment. This is likely related to longer exposure time, greater surface area, and a more stable depositional context, which would facilitate secondary colonization by soil-, water-, and plant-associated fungi.

In contrast, the detached limb harbored fewer unique taxa, such as *M. flavus* and *Penicillium griseofulvum*, indicating more limited or transient environmental contact. The absence of several soil- and plant-associated species on the limb may reflect its more dynamic post-separation history, including relocation by animal activity, which could reduce prolonged contact with a single substrate and limit opportunities for secondary colonization. Some of the taxa found on the detached limb may have originated from the oral cavity of the dog. Vertebrate scavengers are known to complicate the interpretation of forensic evidence (Lira et al. 2020). Although there is no direct evidence that vertebrate scavengers act as vectors of microorganisms, scavenging insects have been shown to facilitate microbial transfer between decomposing remains and the surrounding environment (Deel et al. 2022). Therefore, animal activity may have contributed to changes in postmortem microbial communities.

These findings highlight the importance of extending sampling strategies beyond the human remains themselves. Swabs collected from the surrounding soil, water, vegetation, and potential animal vectors could have significantly improved the interpretation of postmortem fungal communities. Here, lack of such samples is the major limitation of present studies. However, such an approach would allow for a more accurate reconstruction of fungal transfer pathways and strengthen the evidential value of mycological data in forensic investigations.

### Forensic potential of postmortem fungal community profiling

However, the presence of partially preserved fungal signatures across spatially separated remains suggests that mycological community profiling may provide an additional line of evidence for associating detached body parts with their source. Seven out of 23 fungal species (*A. album*,* D. ullevolea*,* Fusarium solani*,* F. aquaeductuum*,* G. candidum*,* M. hiemalis*, and *Y. lipolytica*) were identified on both the cadaver and the separated right upper limb. Fungi involved in the decomposition of dead tissue are typically common saprotrophs (Piepenbring et al. 2025). Similarly, most of the common species are consistent with the regional profile of fungal communities in temperate climates (Madzak 2021; Pottier et al. 2008; Šišić et al. 2018). Additionally, *F. solani*, *F. aquaeductuum*,* G. candidum*, *M. hiemalis*, and *Y. lipolytica* have been reported from human cadavers (Piepenbring et al. 2025; Beaulieu et al. 2025; Spychała et al. 2026).

Currently, the identification of dismembered or isolated body parts relies primarily on nuclear DNA profiling based on short tandem repeats (STR) (Jobling and Gill 2004). Despite its high discriminatory power, STR-based DNA profiling may be limited in certain forensic scenarios, including advanced decomposition or microbial activity can lead to DNA degradation (Bhoyar et al. 2024). Under such conditions, supplementary lines of evidence may be valuable to support forensic conclusions. In this context, microbial evidence has gained increasing attention. Previous studies have demonstrated that microbial communities can be used to link individuals to personal objects through the so-called touch microbiome (Kodama et al. 2019).

To date, however, the application of micromycetes to the association of separated human body parts has not been explored. The results of the present study provide preliminary evidence that fungal community signatures may contribute to this process. Nevertheless, further studies involving a larger number of cases and environmental reference sampling are required to validate this approach and to better understand the relative contributions of endogenous and exogenous fungi in postmortem contexts.

## Conclusion

The study characterized the regional postmortem mycobiome of corpses and a separated upper limb in an advanced state of decomposition in a temperate climate. The research expanded knowledge about skin fungi colonizing human corpses, including the first identification of 11 new species of fungi on human remains. Additionally, the use of fungal profiles as indicators for identifying dismembered body parts of corpses has been considered. Nevertheless, to validate this concept and increase statistical power, it is necessary to expand the number of case studies. Another issue that needs to be addressed is the exclusion of taxa originating from the environment, leaving only those strictly associated with the body surface. Therefore, future studies should include additional environmental samples.

## Supplementary Information

Below is the link to the electronic supplementary material.

ESM1 (PDF 309 KB)

## Data Availability

All data generated or analyzed during this study are included in this article.
