# Supplementary material for: Fungal signatures in a cadaver and a separated upper limb: insights from a forensic case study
Source: Appl Microbiol Biotechnol. 2026 Jun 4;110:237. doi: 10.1007/s00253-026-13895-x (PMC13454014; doi:10.1007/s00253-026-13895-x)
Supplement: Supplementary file 1 [file 253_2026_13895_MOESM1_ESM.pdf]

Supplementary material

Fungal signatures in a cadaver and a separated upper limb: insights from a forensic case study

Applied Microbiology and Biotechnology

Klaudyna Spychała*, Łukasz Szleszkowski, Agata Thannhäuser, Agata Piecuch, Jakub Suchodolski, Rafał Ogórek

*corresponding author: klaudyna.spychala@uwr.edu.pl, Department of Mycology and Genetics, Faculty of Biological Sciences, University of Wrocław, Przybyszewskiego 63, Wroclaw PL-51-148, Poland, https://orcid.org/0000-0002-3135-1386

Autopsy findings

The body exhibited advanced late-stage putrefaction and adipocere formation of the skin surfaces, with extensive soft-tissue loss, and partial skeletonization involving the head, neck, and upper portion of the thorax. Numerous plant leaves and twigs, as well as insects, were present on the remains. The skin was gray-brown, dull, rough, with a finely granular texture; it was hardened, covered with putrid exudate, and detached from the underlying bones. A preserved skin flap on the left upper arm also showed a fine-grained texture but appeared whitish, brittle, and dull, with a gray-white pasty material on its inner surface. Extensive and deep defects reaching the bone were present within the skin and soft tissues, including the neck, nape, shoulders, upper anterior thorax, lateral trunk, and the lower limbs, as well as the left upper limb. The left humerus was connected to the torso only by gray-whitish, loose, macerating ligaments and remnants of joint capsules at the shoulder and elbow joints. The right upper limb, detached from the body, showed a similar pattern, with partial skeletonization (particularly of the arm), while the finely granular skin texture remained intact over the wrist and hand. The cranial cavity contained shrunken, transformed, brown, clay-like brain tissue. The pleural cavities and the abdominal cavity were filled with large amounts of pasty, amorphous, gray-green and greenish-yellow putrid material. The internal organs were in an advanced stage of decomposition, forming a conglomerate in which only a few severely postmortem-altered, flattened anatomical structures could be distinguished. The muscles of the lower limbs were gray-brown-pink, with obliterated fiber architecture, markedly macerated, in places almost pasty, structurally obliterated, and disintegrating. Additionally, traumatic changes consistent with perimortem trauma were identified: a fracture of the right nasal bone with separation of a triangular bone fragment, and a linear fracture of the left parietal bone. In the distal one-third of the right ulna, evidence of a healed fracture consistent with antemortem trauma was noted. Due to the location where the body was found (a roadside ditch), the autopsy technique was extended to include deep dissection of the lower limbs (bones and joints). No injuries were found that would be typical of pedestrian impact in an upright position or of being run over while lying down. Among pathological changes, only degenerative-proliferative alterations of the spine were identified.
